# Big data for bipolar disorder

**DOI:** 10.1186/s40345-016-0051-7

**Published:** 2016-04-11

**Authors:** Scott Monteith, Tasha Glenn, John Geddes, Peter C. Whybrow, Michael Bauer

**Affiliations:** Michigan State University College of Human Medicine, Traverse City Campus, 1400 Medical Campus Drive, Traverse City, MI 49684 USA; ChronoRecord Association, Inc, Fullerton, CA 92834 USA; Department of Psychiatry, Warneford Hospital, University of Oxford, Oxford, OX3 7JX UK; Department of Psychiatry and Biobehavioral Sciences, Semel Institute for Neuroscience and Human Behavior University of California Los Angeles (UCLA), 300 UCLA Medical Plaza, Los Angeles, CA 90095 USA; Department of Psychiatry and Psychotherapy, Universitätsklinikum Carl Gustav Carus, Technische Universität Dresden, Fetscherstr. 74, 01307 Dresden, Germany

**Keywords:** Bipolar disorder, Big data, EMR, Registries, Claims, Patient monitoring

## Abstract

The delivery of psychiatric care is changing with a new emphasis on integrated care, preventative measures, population health, and the biological basis of disease. Fundamental to this transformation are big data and advances in the ability to analyze these data. The impact of big data on the routine treatment of bipolar disorder today and in the near future is discussed, with examples that relate to health policy, the discovery of new associations, and the study of rare events. The primary sources of big data today are electronic medical records (EMR), claims, and registry data from providers and payers. In the near future, data created by patients from active monitoring, passive monitoring of Internet and smartphone activities, and from sensors may be integrated with the EMR. Diverse data sources from outside of medicine, such as government financial data, will be linked for research. Over the long term, genetic and imaging data will be integrated with the EMR, and there will be more emphasis on predictive models. Many technical challenges remain when analyzing big data that relates to size, heterogeneity, complexity, and unstructured text data in the EMR. Human judgement and subject matter expertise are critical parts of big data analysis, and the active participation of psychiatrists is needed throughout the analytical process.

## Background

The frequency and importance of comorbid mental and chronic physical illness have emphasized the need for a change in the delivery of psychiatric care, including bipolar disorder (Melek et al. 2014, DeHert et al. 2011). Bipolar disorder is associated with poor functional outcome (Conus et al. 2014), considerable economic cost for society (Kleine-Budde et al. 2014; Young et al. 2011), and management is often complicated by medical comorbidity such as type II diabetes/insulin resistance (Calkin et al. 2015; Calkin and Alda 2015; Carney and Jones 2006). Responses to improve care delivery include integrating psychiatry with primary care (Butler et al. 2008; Manderscheid and Kathol 2014; Cerimele and Strain 2010; Katon et al. 2010), collaborative care measures (Woltmann et al. 2012), implementing preventive programs and quality measurements consistent with a population health perspective (Rose 2001; Mabry et al. 2008), and increasing emphasis on the genetic and neuroscience basis of mental illness (Insel 2009; Reynolds et al. 2009). Additionally, precision medicine initiatives are accelerating interdisciplinary research with a goal of tailoring psychiatric care to the individual (Insel 2014).

Big data and advances in the ability to analyze these data are fundamental to this evolving perspective of psychiatry (Monteith et al. 2015; NRC 2013). Big data can be conceptualized as heterogeneous data, unprecedented in size and complexity, lacking in structure, and coming from many sources (Monteith et al. 2015). The scale of big data in size and complexity makes it difficult to process, analyze, and extract useful information (Burkhardt 2014). Today, the primary source of big data in medicine is from providers and payers including electronic medical records (EMR) created by physicians, claims records, pharmacy records, and imaging. However, the data for analysis will keep expanding from omics, such as genomic, epigenomic, proteomic, and metabolomic data. Today, about 95 % of the data for each patient is generated by imaging (Hamalka 2011), and genomic data requires 50-fold greater storage per patient than imaging (Starren et al. 2013). Data will also be coming from non-traditional sources including patients and non-providers, from smartphone applications, sensors, and Internet activities (Glenn and Monteith 2014a). With the addition of data from patient devices, it is estimated that every person will generate more than 1 petabyte (1 million gigabytes) of health information over a lifetime (IBM 2015a). IBM envisions a future in which 10 percent of medical data will be from medical records, 20 percent from genomics, and 70 % from patient-created sources (Slabodkin 2015). The amount of medical-related data in existence is expected to double in size every 2 years (IBM 2015b).

It is still early in the process of converting from paper to digital-based medicine. As with other industries, the main benefits will be related to future innovations and redefined work processes fostered by the technology, and increased software usability and usefulness (Fernald and Wang 2015; Landauer 1995). However, many initial benefits from digitizing data are already being seen today in the analysis of very large databases. The objective of this review is to discuss both the promises and challenges of using big data to improve the understanding and treatment of bipolar disorder.

## Data sources from providers and payers

There are many public and private sources of big data from EMR, claims/administrative data, and registries that are available for secondary use in medical research. These data sources were not designed for research and each has strengths and weaknesses, with differences in quality, completeness, and potential for bias. In the US, claims or administrative encounter data that providers (physicians, hospitals, labs, and pharmacies) submit for payment to insurers and the government provide the most complete picture of patient involvement with the healthcare system. Although standardized diagnostic and procedure codes are used, claims data lacks clinical detail such as test results. The diagnosis on a claim is only for the services performed on that date, and may be incorrect, incomplete, differential, or driven by reimbursement policies (Sarrazin and Rosenthal 2012; Wilson and Bock 2012; West et al. 2014; Overhage and Overhage 2013). The time lag for claims processing is often several months. About 17 % of commercially insured people in the US switch coverage each year posing challenges for longitudinal analysis (Sung 2015; Marketscan 2011).

In contrast to claims, EMR provide timely clinical details from the providers who use the software, especially related to patient management. The clinical data may include patient history and symptoms, multiple diagnoses including those unrelated to the current visit, physician assessment and treatment plan, disease severity, lab results, vital signs, non-prescription drugs and results of screening tools such as PHQ-9. Government mandates in the US have dramatically increased the use of EMR. About half of EMR text is unstructured data (Davenport 2014), and many challenges remain to automatically extract information from the rich but distinct vocabularies used throughout medicine (Dinov 2016; Ivanovic and Budimac 2014). Efforts are underway to address standardization with the goal of semantic interoperability of data from different providers and software systems (IHE 2015; HealthIT.gov 2015; Dinov 2016). There are other important quality issues in EMR data including inconsistency, redundancy, inaccuracy, missing data, interoperability between vendor products, and potential biases from measured and non-measured confounders (Monteith et al. 2015; Bayley et al. 2013; Kaplan et al. 2014; Hersh et al. 2013; Hripcsak et al. 2011).

Outside the US, psychiatric register data may be based on a country population such as in the Nordic countries or Taiwan, or a geographical area such as the South London and Maudsley NHS Foundation Trust (SLAM) case register, or a provider (Munk-Jorgensen et al. 2014; Allebeck 2009; Stewart et al. 2009). These registries provide a longitudinal record of all psychiatric contacts, and have high coverage and low dropout rates in countries with a national health service. However, there are limitations to the validity and quality of data in psychiatric registries, including over-representation of severe cases or inpatient data, sparse clinical detail, exclusion of variables not available from all institutions reporting to the register, and insufficient linking to other registries such as cause of death (Munk-Jørgensen et al. 2014). There are also questions about the validity of psychiatric diagnoses in the register data (Byrne et al. 2005; Øiesvold et al. 2013), including bipolar disorder (Øiesvold et al. 2012). Psychiatric case registries do not include patients without a psychiatric diagnosis for comparison (Munk-Jørgensen et al. 2014). Some other types of registries that can be linked to psychiatric registries include those for general health, prescription drugs, vital statistics, school registries, social insurance registries, and biobanks (Allebeck 2009), each of which has strengths and weaknesses.

Other sources of data include research databases and surveys, such as the US National Comorbidity Survey (Kessler et al. 1994) or the National Epidemiological Survey on Alcohol and Related Conditions (NESARC) (Grant et al. 2004), which may have a national scope but contain a subset of clinical information.

Even very large databases containing millions of individuals may not be representative of the general population (Riley 2009). For example, the US claims/administrative data from a Medicaid population will include more younger women and children, data from an employer-offered HMO may include more younger and healthier people, and data from Veterans Affairs (VA) will include mainly males and be older (Overhage and Overhage 2013; Medicaid 2015). In a US multistate EMR database with 84 million patients, psychiatric and behavioral diagnoses were less frequent as compared to the US National Inpatient Sample, an established population estimate based on claims (HCUP 2015; DeShazo and Hoffman 2015). Population-based registries from small homogenous countries may not be representative of the population in larger diverse countries. Due to the heterogeneity among very large databases, the data source selected may challenge the results of observational studies, including even finding contradictory statistical significance (Madigan et al. 2013; Goldstein and Winkelmayer 2015; Crump et al. 2013a). However, with a clear understanding of the strengths and weaknesses of a database, some findings from observational analyses can now be verified in many national and regional settings. For example, in a systematic review of 25 international population or community-based studies using different diagnostic criteria, the prevalence of bipolar disorder type I and type II was consistently low (Clemente et al. 2015).

The addition of complementary data sources may improve the accuracy and usefulness of data from any one source. Even when using validated algorithms, it is difficult to determine an episodic diagnosis such as depression when analyzing US claims data, and combining another data source such as EMR may improve accuracy (Townsend et al. 2012; Fiest et al. 2014). However, in the US, linking of data from unrelated sources that were de-identified to meet privacy regulations is challenging (West et al. 2014, Li and Shen 2013). In contrast, many European countries have a unique person identifier that is present on all medical data (Allebeck 2009). The use of complementary linked databases may also expand the types of research questions that may be addressed. Examples of useful linkages include register population data linked with biobank data in a study that found no association between markers of prenatal infection and the risk of bipolar disorder (Mortensen et al. 2011), and in a study that found elevated C-reactive protein was associated with an increased risk of late-onset bipolar disorder (Wium-Andersen et al. 2015).

## Uses for data from providers and payers

The analysis of very large databases has provided fundamental information about bipolar disorder including the incidence, prevalence, decreased life expectancy (Munk-Jørgensen et al. 2014; Allebeck 2009; Laursen et al. 2007; Chang et al. 2011; Kessing et al. 2015c; Kessing et al. 2015d), and trends in prescribing medication (Baldessarini et al. 2007; Hayes et al. 2011; Bjorklund et al. 2015). Results from the analysis of large data sources are continuously being incorporated into patient care and research, and some key areas are discussed below.

### Health policy decisions

Health policy decisions focus on outcome and cost. Big data is fundamental to the increasing importance of clinical guidelines, defining and measuring metrics that reflect the quality of care delivered, and meeting performance standards based on quality metrics. For the treatment of bipolar disorder, big data studies are helping to characterize problems and evaluate the results of policy changes. Of great concern are repeated findings of excess mortality in patients with bipolar disorder due primarily to physical illness, and of continuing disparities in the treatment of physical illness as compared with the general population (Roshanaei-Moghaddam and Katon 2009; McGinty et al. 2015). Some examples of suboptimal care for medical illness for people with bipolar disorder found using big data are shown in Table 1. In addition to health services and physical illness, socioeconomic factors and patient behaviors contribute to excess morbidity and mortality in bipolar disorder (Druss et al. 2011). The linking of psychiatric data with other databases, such as government financial databases, will help to clarify the complex, cumulative impacts of diverse socioeconomic factors, as shown in Table 2. Examples of studies directly related to health policy and bipolar disorder using big data are given in Table 3.

**Table 1:** Examples of studies suggesting suboptimal treatment of medical illness in bipolar disorder

Country	Description	Primary finding	Data source	Number of subjects analyzed (N)	Reference
Denmark	Investigate cardiovascular (CV) drug use and the excess mortality in BP and schizophrenia (SCZ)	Under-prescription of most CV drugs to patients with BP or SCZ compared to general population	Population registries during 1995–1996 of those who used CV drugs	254 with BP, 609 with SCZ, 23,065 with no mental illness	Laursen et al. 2014
Denmark	Investigate hospital contact for CV disease by patients with BP or SCZ compared with general population	Despite excess mortality, rates of contact for those with BP or SCZ similar to general population and lower rates of invasive procedures	Register data from 1994 to 2007	4997 with heart disease and BP or SCZ, 566,071 with heart disease and no mental illness	Laursen et al. 2009
Scotland	Investigation of medical comorbidities in BP	Frequent wide ranging medical comorbidities. CV disease under-recognized and undertreated	Primary care registry for about 1/3 of Scottish population in 2007	2582 with BP and 1,421,796 without	Smith et al. 2013
Sweden	Estimate CV mortality in BP compared to general population	Mortality rate ratios for CV disease twice as high for BP than general population. People with BP died of CV disease about 10 years earlier than general population	National population register 1987–2006	17,101 patients diagnosed with BP in general population of 10.6 million	Westman et al. 2013
Sweden	Impact of physical health on mortality rate in BP	Frequent premature mortality is from chronic medical diseases. However, mortality from chronic diseases among those with prompt treatment approached that of general population	National population registries between 2001 and 2002, with follow-up 2003–2009	6618 diagnosed with BP	Crump et al. 2013b
Taiwan	Use of invasive diagnostic and revascularization procedures after acute myocardial infarction (AMI) in patients with SCZ or BP	Patients with BP and SCZ half as likely to receive catheterization or revascularization procedures after AMI	National register from 1996 to 2007	3661 patients with AMI of which 591 with SCZ and 243 with BP	Wu et al. 2013
UK	Compare screening for CV risk in primary care of patients with SCZ or BP to patients with diabetes	Much less screening of patients with mental illness for CV risk (1/5 versus 96 %)	Five primary care centers in Northampton, England	368 with mental illness; 1875 with diabetes	Hardy et al. 2013
UK	Compare screening for metabolic risk in primary care of patients with SCZ or BP to patients with diabetes	Less screening of patients with mental illness for metabolic risk (74.7 versus 97.3 %)	NHS database between 2010 and 2011	2,488,948 patients with diabetes and 422,966 patients with mental illness	Mitchell and Hardy 2013
US	Impact of guidelines released by American Diabetic Association (ADA) in 2004 on glucose monitoring in patients treated with second generation antipsychotics (SGA)	Low levels of monitoring despite small improvement after guidelines (just over 10 % lipid monitoring; just over 20 % glucose monitoring)	Managed care database of patients under age 65 between 2000 and 2006	5787 patients before guidelines; 17,832 after	Haupt et al. 2009
US	Investigate diabetes screening in patients with SCZ and BP who take antipsychotics over a 1 year period	Almost 70 % not screened for diabetes using validated screening measures. Those with at least one primary care visit more than twice as likely to be screened	CA Medicaid population during 1/2009–12/2009, and 10/2010–10/2011	50,915 patients with SCZ, BP and other severe mental illness	Mangurian et al. 2015
US	Investigate hospitals selected for patients with mental illness and acute myocardial infarction (AMI)	Comorbid mental illness was associated with an increased risk for admission to lower-quality hospitals. Both lower-quality hospital and mental illness predicted worse outcome	Medicare population in 2008, aged ≥65 years	287,881 patients with AMI, of which 41,044 also with mental illness	Cai and Li 2013

**Table 2:** Examples of big data studies of socioeconomic factors in bipolar disorder

Country	Description	Primary finding	Data source	Number of subjects analyzed (N)	Reference
Denmark	Association of BP and schizophrenia (SCZ) with parent–child separation	Associations found but differed by type, developmental timing and family characteristics	Danish register between 1971 and 1991, followed to 2011	2821 with BP and 6469 with SCZ	Paksarian et al. 2015
Denmark	Association between mortality and lifetime substance use disorder in patients with BP, SCZ or unipolar depression	Mortality in people with mental illness far higher for those with substance use disorders; especially involving alcohol or hard drugs	Those born in Denmark in 1995 or later	41,470 with SCZ, 11,739 with BP, and 88,270 with unipolar depression	Hjorthoj et al. 2015
Israel	Percentage of patients with BP and SCZ and other psychosis, who earn at least minimum wage	For BP: with 1 hospital admission, only 24.2 % earned at least minimum wage; with multiple admissions, 19.9 %. Poor employment outcome in all cases	Israeli psychiatric hospitalization registry	35,673 total	Davidson et al. 2015
Sweden	Compare risks for suicidality and criminality in patients with BP and general population	22.2 % of BP engaged in suicidal or criminal acts after diagnosis. Combined risk of suicidality and criminality is elevated	Swedish national registries between 1973 and 2009	15,337 with BP, compared with 14,677 unaffected siblings	Webb et al. 2014
Sweden	Association of high intelligence and BP	High intelligence may be a risk factor for BP, but only in those without psychiatric comorbidity	Diagnosis of BP from Hospital Discharge Register from 1968 and 2004. IQ measure at military conscription	1,049,607 males. 3174 hospitalized with BP	Gale et al. 2013
Sweden	Association of leadership traits with BP	Traits associated with BP may be linked to superior leadership qualities	Swedish population registries from 1973 and 2009	68,915 with BP, and healthy siblings	Kyaga et al. 2015
Sweden	Investigate disease burden in bipolar disorder	Compared to general population, patients had same education, more unemployment, less disposable income, and twice the mortality	Swedish population registries of all diagnosed with BP 1991–2010; cohort in 2006 versus 2009	4629 in 2006; 5644 in 2009	Carlborg et al. 2015
US	Association of BP and SCZ with criminal justice involvement	Males and females with BP disorder have higher risk for offending than those with SCZ; highest risk is BP plus substance use disorder	Connecticut mental health administrative records plus criminal justice records	25,133 adults, 5479 with BP and substance abuse; 7327 with BP alone	Robertson et al. 2014
US	Employment and functional limitations in BP and unipolar depressive disorders	Patients with BP significantly more unemployment and functional limitations than those with depressive disorders or controls	Nationally representative Medical Expenditure Panel survey 2004–2006	592 with BP, 5646 with depressive disorders, 53,905 controls	Shippee et al. 2011
UK	Childhood IQ and risk of BP	Higher childhood IQ may be a marker for risk of later BP	Avon birth cohort. IQ at age 8; lifetime manic features at age 22–23	1881 individuals	Smith et al. 2015

**Table 3:** Examples of big data projects related to health policy for patients with bipolar disorder

Country	Description	Primary finding	Data source	Number of subjects analyzed (N)	Reference
France	Impact of longitudinal continuity of care with the same community psychiatrist on mortality rate of patients with mental disorders	Higher the continuity of care the lower likelihood of death, especially in those with BP, major depressive disorder and schizophrenia (SCZ)	France national claims data 2007–2010	14,515 patients visiting psychiatrist at least once, tracked over 3 years	Hoertel et al. 2014
UK	Investigation of delay between first visit to a mental health service and a diagnosis of BP	Median diagnostic delay was 62 days; median treatment delay was 31 days	SLAM register data between 2007 and 2012	1364 diagnosed with BP	Patel et al. 2015b
UK	Investigation of mortality after hospital discharge with principal diagnosis of BP or SCZ	Standardized mortality ratios about double general population. For BP, increased from 1.3 in 1999 to 1.9 in 2006. About 3/4 of all deaths from natural causes	English national hospital and death registries from 1999 and 2006	100,851 hospital discharges for patients with BP and 272,248 with SCZ	Hoang et al. 2011
US	Impact of state Medicaid formulary restrictions on total medical costs for patients with BP or SCZ	Medication adherence declined due to formulary restrictions. Total medical costs increased	Medicaid claims from 24 states 2001–2008	170,596 patients with BP and 117,908 with SCZ	Seabury et al. 2014
US	Impact of requiring prior authorization (PA) for more expensive medications on the discontinuation of antipsychotics and anticonvulsants	Higher rates of discontinuation of all medication treatment. No increase in use of preferred drugs (not requiring PA)	Medicaid and Medicare claims 2001–2004 in Maine	N = 5336 Maine N = 1376 New Hampshire (comparison state)	Zhang et al. 2009
US	Impact of prior authorization and copayments policy on medication continuity	Prior authorization and copayments decreased medication continuity. (High continuity in 54 % of those with BP and 64 % of those with SCZ)	Medicaid claims from 22 states in 2007	33,234 patients with BP and 91,451 with SCZ	Brown et al. 2013
US	Impact of adherence to and persistence with atypical antipsychotics on health care costs	Good adherence and persistence led to lower costs	Commercial health insurance claims 2007–2013	32,374 patients with diagnosis of BP or SCZ and prescription for oral antipsychotic	Jiang and Ni 2015
US	Association of frequent psychiatric interventions over 1 year on health care utilization and costs in patients with BP I	Patients needing frequent psychiatric interventions had higher psychiatric and general medical utilization and costs in following year	Commercial insurance claims 2004–2007	7260 patients with frequent psychiatric interventions and 11,571 without	Bagalman et al. 2011
US	Examine conformance to practice guidelines for children/adolescents with BP	Most received recommended therapy but only a minority received drug monitoring and/or recommended psychotherapy	Medicaid in Ohio 2006–2010	4047 youths aged 15–18 years with new episode of BP	Fontanella et al. 2015
US	Estimate number of emergency department (ED) visits by adults involving psychiatric medications	Antipsychotics and lithium involved in more visits relative to rate at which prescribed. Half of ED visits involving psychiatric medications were for patients 19–44 years	National surveillance database from 63 hospitals between 2009 and 2011	89,094 ED visits annually for therapeutic use of psychiatric medications in patients ≥19 years	Hampton et al. 2014
US	Evaluate if patients with SCZ and BP received comprehensive treatment by state	In each state, only 45 % with BP, and 47 % with SCZ had a continuous medication supply. About 25 % of beneficiaries had no mental health visit	Medicaid claims in 21 states + DC in 2007	40,609 with BP; 102,884 with SCZ	Brown et al. 2015
US	Drug utilization patterns for newly initiated atypical antipsychotic	Low adherence and persistence: 63.4 % discontinued index therapy, and majority of these (69.5 %) did not resume any antipsychotic	Commercial insurance between 2002 and 2008	16,807 patients ≥18 years with BP I	Chen et al. 2013

### Evaluation of rare events

Big data allows the study of rare events and outcomes that may require data from multiple sources to provide an adequate sample size for detection. Randomized controlled trials are not powered to detect rare events or long-term effects, and case control and retrospective cohort study designs of observational databases collected from clinical practice are often used (Chan et al. 2015; Rodriguez et al. 2001). For example, there have been several recent large or population-based studies of renal related events in patients who were treated with lithium, as shown in Table 4. Big databases are being used for pharmacovigilance of many drugs prescribed for bipolar disorder, such as studies of the potential for antipsychotics to increase risk of a seizure (Bloechliger et al. 2015), pulmonary embolism (Tournier 2015; Conti et al. 2015), and a Torsades de pointes ventricular arrhythmia (Poluzzi et al. 2013).

**Table 4:** Examples of big data projects related to lithium and renal function

Country	Description	Primary finding	Data source	Number of subjects analyzed (N)	Reference
Denmark	Examine association between long-term lithium use (≥5 years) and risk of renal and upper urinary tract cancers	Not associated with an increased risk	Danish Cancer Registry between 2000 and 2012	6447 cases matched to 259,080 controls	Pottegard et al. 2016
Denmark	Compare rates of chronic kidney disease (CKD) and end-stage CKD in patients taking lithium or other drugs for BP	Maintenance treatment with lithium or anticonvulsants increases rate of CKD, but lithium is not associated with increased rate of end-stage CKD	Danish population registries 1994–2012	1,500,000 randomly selected controls, 26,731 exposed to lithium and 420,959 to anticonvulsants for any reason. 10,591 with primary diagnosis of BP	Kessing et al. 2015a
Denmark	Assess risk of renal and upper urinary tract tumors among lithium users	Not associated with an increased risk	Danish population registries 1995–2012	1,500,000 randomly selected controls, 24,272 exposed to lithium and 386,255 to anticonvulsants for any reason. 9651 with primary diagnosis of BP	Kessing et al. 2015b
Italy	Examined glomerular filtration rate (GFR) in patients with long-term lithium treatment	Lithium is a risk factor for reduced GFR. Renal dysfunction tends to appear after decades of treatment and to progress slowly. Median time to enter G3a was 25 years	Lithium register from 1980 to 2012	953 patients. Patients treated up to 33 years	Bocchetta et al. 2015
Scotland	Comparison of estimated glomerular filtration rate (eGFR) in patients recently started on lithium therapy versus those taking other medications for affective disorders	No effect of stable lithium maintenance therapy, with lithium levels in the therapeutic range, on rate of change in eGFR over time	Population of patients started on lithium therapy in Tayside between 2000 and 2011	305 in lithium group; 815 in comparator group. Mean duration of exposure 55 months	Clos et al. 2015
Sweden	Determine prevalence and extent of kidney damage during course of long-term lithium treatment	About one-third of patients treated for ≥10 years had evidence of chronic renal failure; only 5 % severe. Continuous monitoring of kidney function is required	Lab data from all Gothenburg area public hospitals and clinics	630 patients starting lithium after 1980 with ≥10 years of cumulative lithium treatment	Aiff et al. 2015
UK	Compared lab measures of renal, thyroid and parathyroid function in those with at least two lithium measurements versus those with no lithium measurements	Lithium treatment associated with decline in renal function, hypothyroidism and hypercalcemia. Women <60 years with lithium concentrations higher than median at greatest risk. Long-term monitoring needed	Lab data from Oxfordshire area between 1985 and 2014	2795 ≥18 years with at least two lithium measurements; 689,228 controls	Shine et al. 2015
UK	Assess association between lithium use and renal failure in patients with bipolar disorder	Ever use of lithium was associated with an increased risk of renal failure (adjusted hazard ratio 2.5). Absolute risk of renal failure was age dependent and small	General practice research database from 418 practices between 1990 and 2007	6360 with BP; 2496 lithium users; 3864 non-users	Close et al. 2014
US	Possibility of stratifying risk for renal insufficiency among lithium treated patients	Use of lithium more than once daily; lithium levels >0.6 mEq/l, and use of first generation AP independently associated with risk	EMR records from large healthcare system 2006–2013	1445 lithium users with renal insufficiency; 4306 lithium users for comparison	Castro et al. 2015b

### Exploration and hypothesis generation from large databases

The exploration of big data offers unique opportunities to find correlations that may trigger the investigation of new areas and generation of new hypotheses (Varian 2014; Khoury and Ioannidis 2014). These new correlations may or may not have meaning, do not measure causality, and may be further investigated by traditional or data-intensive experimental methods as appropriate. There are many computational and statistical challenges associated with the analysis of big data related to the number of patients, number of variables per patient, and the quality and technical complexity of the databases (Monteith et al. 2015; Fan et al. 2014; Grimes and Schulz 2002). Both the variables included and the analytic techniques used may lead to variation in the associations detected in big data studies (Abrams et al. 2008; Fan et al. 2014; Patel et al. 2015a).

Additional correlations detected include an association between epilepsy and bipolar disorder (Wotton and Goldacre 2014; Clarke et al. 2012), an increased risk of pneumonia in patients with bipolar disorder taking antipsychotics (Yang et al. 2013), an increased risk of bipolar disorder in those with a diagnosis of autism spectrum disorder (Selten et al. 2015), and finding that the premature risk of cardiovascular disease in bipolar disorder is not explained by traditional risk factors including cigarette smoking, obesity, or hypertension (Goldstein et al. 2015). In a study using medical records from 110 million patients, new associations were found between Mendelian diseases and complex psychiatric diseases, including bipolar disorder (Blair et al. 2013).

### Defining phenotypes

There is considerable interest in using EMR to automate the process of defining phenotypic cohorts for genetic studies of bipolar disorder, since sample sizes of tens of thousands are needed (Pathak et al. 2013; Potash 2015). In addition to the study of phenotype-genotype relationships and gene-disease associations, phenotypic cohorts will enable a wide range of clinical research. Despite many challenges, semi-automated methods are now being used to define phenotypes from EMR for psychiatric disorders, including bipolar disorder (Lyalina et al. 2013; Castro et al. 2015a). The methodology used to automate phenotype detection in EMR is evolving, and includes data mining, natural language processing, statistical techniques, and human expertise (Hripcsak and Albers 2013; Pathak et al. 2013). More standardization is expected in the future.

### Predictive models

Predictive models are widely used in medicine, such as cardiovascular risk prediction, to estimate the presence of a diagnosis or event, or if the diagnosis or event will occur in a specific time period (Moons et al. 2012). The results of validated predictive models may assist the physician and patient with decision making to mitigate risks, and help to limit spending on unnecessary procedures. Before adoption for clinical use, predictive models require considerable testing and re-adjustment, including internal validation, external validation with other populations, followed by determination if the validated model provides actionable information to the clinician and patient (Moons et al. 2012). Most predictive models are based on a small number of variables collected in cohort studies such as the Framingham Heart Study (D’Agostino et al. 2008). In general, models used in medicine today have limited predictive power, and access to the large number of variables and patients in EMR and other databases may improve their accuracy in the future (Berger and Doban 2014; de Lissovoy 2013). With the frequent use of heuristics in medical decision making, complex predictive models also need practical input requirements for routine use in clinical situations (Marewski and Gigerenzer 2012).

Many technical issues impede the development of predictive models from EMR data, including quality, multidimensional complexity, bias, comorbidities, and confounding medical interventions (Paxton et al. 2013; Wu et al. 2010; Wang et al. 2014). The temporal nature of EMR data also poses a significant challenge for prediction (Singh et al. 2015; Binder and Blettner 2015). In contrast to a controlled longitudinal study, data entries into an EMR only occur when a patient initiates or a physician recommends and documents care. There are great differences in the time between visits for one patient, and across all patients, in the number of visits and length of time each patient is tracked. New variables detected in EMR data may be associated with but not predictive of disease (Ware 2006). A variety of machine learning, data mining, classification algorithms, and statistical approaches are currently being researched for the future (Singh et al. 2015; Wu et al. 2010, Wang et al. 2014).

While the primary benefits of prediction will be in the future, in some recently developed models, bipolar disorder is a risk factor for readmission to a psychiatric hospital within 30 days of discharge (Vigod et al. 2015), readmission to a safety-net hospital within a year (Hamilton et al. 2015), and suicide by veterans (McCarthy et al. 2015). The addition of variables relating to a diagnosis of bipolar disorder or schizophrenia improved the accuracy of a predictive model of cardiovascular risk for those with these diagnoses (Osborn et al. 2015).

## Data sources from patients and non-providers

Digital technologies that are widely accepted by the general public are being integrated into the routine care of bipolar disorder to increase patient involvement and expand clinician oversight between visits. Many technologies are suitable platforms for active or passive patient monitoring including computers, smartphones, and even clothing with embedded sensors. Today, the patient-created data are not generally integrated into the EMR.

### Data actively created by patients outside of medical settings

Many applications are available today to monitor bipolar disorder away from medical settings that require active patient participation. These include validated products for mood charting such as the ChronoRecord on a computer (Bauer et al. 2004; Bauer et al. 2008), the Life-Chart on a smartphone and web site (Scharer et al. 2015), weekly text messaging of responses to Quick Inventory of Depressive Symptomatology and Altman self-rating manic scale (Bopp et al. 2010), and weekly or monthly use of an interactive voice response (IVR) system to complete the PHQ-9 (Piette et al. 2013). In all cases, the patients respond to questions or prompts directly related to their illness. In addition to clinical use, data collected from these systems is often aggregated for research (Bauer et al. 2013a, 2013b; Moore et al. 2014). A large number of parameters may be accumulated for each patient, such as from daily medications taken (Bauer et al. 2013a), but data are not routinely integrated into the EMR. Although challenges remain regarding the interpretation of self-reported data, much of the understanding about the long-term course of bipolar disorder is due to the daily recording efforts of patients worldwide, starting with paper-based instruments (Bauer et al. 1991; Kupka et al. 2007).

### Data passively created by patients outside of medical settings

With passive monitoring, patients do not directly provide information about their illness, and much of the data collected are non-medical. For example, data from Internet and smartphone activities, and from sensors in smartphones and wearable technology, are routinely being used to monitor mental state and behavior for non-medical purposes such as behavioral advertising (Glenn and Monteith 2014b; Geller 2014; FTC 2009). There are a variety of passive monitoring projects for bipolar disorder, mostly in the pilot phase, with examples shown in Table 5. The implementation of routine passive monitoring for large numbers of patients faces many hurdles, including patient acceptance, physician usability, and processing large volumes of data from sensors (Redmond et al. 2014; Muench 2014). Many passive monitoring projects involve smartphones. Both the differing physical characteristics of the standard devices available to consumers such as sensor accuracy and memory size, and methods selected for analysis may impact the findings (Banaee et al. 2013; Redmond et al. 2014). The sales of smartphones are flat in developed countries with saturation reached, and usage patterns vary among countries (Thomas 2014, Waters 2015). In the US in 2015, 64 % of adults in the US use a smartphone with 7 % relying primarily on it for Internet access (Smith 2015).

**Table 5:** Examples of passive monitoring of patients with bipolar disorder related to smartphones, Internet activities, or wearables

Technology	Sensors	Aim	Primary measures	N	Findings	Study
Ingestible^a^	Ingestible sensor in tablets. Wearable sensor on torso	Measure medication adherence	Adherence metrics. Logs date and time of tablet ingestion	28	System is feasible in patients with BP and SCZ	Kane et al. 2013
Internet social media		Differentiate depression subgroups by language use	Analyze topics and linguistic features in 24 online communities interested in depression	5000 blog posts	Five distinct subgroups, one is BP. For those with BP, topics on medications and BP most important	Nguyen et al. 2015
Internet social media		Explore language differences among 10 mental health conditions	Using public Twitter posts 2008–2015, group by classifiers including self-reported diagnosis	>100 users/group; >100 posts/user	Language usage patterns differ by condition	Coppersmith et al. 2015
Smartphone	Accelerometer, GPS	Detect mood state	Daily mobility (physical motion), and travel patterns (number of locations visited, time outdoors)	12	Can detect a change in mood state. Less precise to detect mood state	Gruenerbl et al. 2014
Smartphone	Accelerometer; microphone	Detect mood state	Number of apps running; app usage patterns and selection. MONARCA software	18	Patterns of app usage vary with self-reported mood	Alvarez-Lozano et al. 2014
Smartphone	Accelerometer	Detect mood state	Overall activity levels	9	Substantial individual variation in activity levels, both daily and within intervals	Osmani et al. 2013
Smartphone		Detect mood state	Number and duration of ingoing and outgoing calls; number of text messages. MONARCA software	61	Patterns of calls and texts vary in manic and depressive mood states	Faurholt-Jepsen et al. 2015
Smartphone	Microphone	Detect mood state	Phone call statistics; acoustic emotional analysis, and social signals from daily calls	12	Speaking length and call length among the most important predictors of mood	Muaremi et al. 2014
Smartphone	Recorder for outgoing speech	Detect mood state	Voice monitoring and acoustic analysis of speech patterns from continuously recorded outgoing calls	6	Can recognize manic and depressive mood states	Karam et al. 2014
Wearable (T-shirts)	Electrodes and sensors integrated into garment	Detect mood state	ECG and respiration. Long term heart rate variability analysis. PSYCHE monitoring system	8	Can differentiate mood states (depressed, manic, mixed, euthymic)	Valenza et al. 2014

^a^ New drug application submitted to FDA by Otsuka pharmaceuticals and proteus digital health for sensor-embedded Abilify in September, 2015

### Commercial processing of data

Provider-created data are traditionally processed by the provider or their contractors. In contrast, commercial firms unrelated to medicine may be involved in both active and passive patient monitoring. Many behavioral related apps are available for Apple and Android smartphones, and commercial firms may receive, store, and analyze data using proprietary and unvalidated algorithms. Any potential combination of data processed by commercial firms with EMR data needs to be carefully evaluated as the firms may not be covered by national privacy regulations (Glenn and Monteith 2014b). An analysis of 79 mobile health apps certified as trustworthy by the UK NHS found a multitude of privacy and security flaws (Huckvale et al. 2015).

### Changing world of technology

Passive monitoring should be considered in the context of the ongoing changes in digital technology, especially in relation to mobile devices for consumers. First, the devices used to access the Internet will change the online activities of the public. For example, the use of a search engine is much lower from a smartphone than from a computer (Arthur 2015; MacMillan 2015). Second, the widespread use of mobile technology has triggered a push toward developing artificial intelligence (AI) interfaces for devices, as evidenced by the near simultaneous announcements of open source AI software tools from Google, Microsoft, IBM, and Facebook (Simonite 2015). The vision of Larry Page of Google is for Google to tell you what you want before you ask the question (Varian 2014, Page 2013). In an international survey of 6600 smartphone users by Ericsson, half of all smartphone users expect AI interfaces to replace the smartphone screen within 5 years, and one-third want AI to keep them company (Boulden 2015). Messaging chatbots (computer-generated responses based on AI) are starting to replace search engines on mobile devices (Elgan 2015). In the future, consumer mobile devices will routinely incorporate voice and gesture input, and as hardware features change, the AI algorithms will also evolve. In the background, there is an industry-wide effort to develop AI algorithms based on massive databases to predict behavior and emotions for uses such as for targeted marketing.

## Other provider data sources

Massive amounts of data will be coming from genomics, proteomics, and image processing, and the ongoing efforts of large-scale consortia will help to elucidate the neuropathology of bipolar disorder and define new treatment targets. The ENIGMA Consortium detected subcortical brain volumetric changes using brain structural MRI scans from 1710 patients with bipolar disorder and 2594 controls (Thompson et al. 2014, Hibar et al. 2016). The ConLiGen Consortium identified genetic variants associated with lithium response in a GWAS study of 2563 patients with bipolar disorder (Hou et al. 2016). The Psychiatric Genomics Consortium (PGC) found a new susceptibility locus in a GWAS study of 7481 individuals with bipolar disorder and 9250 controls (Sklar et al. 2011). Recent technology allows large-scale comparison of proteome profiles (Gold et al. 2010; SomaLogic 2016), and findings may improve predictive models for bipolar disorder. These data are not expected to be incorporated into the EMR or impact the routine care of bipolar disorder in the near future but suggest future directions for data integration.

## General considerations

There are a wide range of anticipated and unanticipated complications related to the use of big data in the study of bipolar disorder some of which are mentioned briefly below.

### Privacy and confidentiality

The privacy and confidentiality of big data are a major concern. Many technical issues affect the privacy and confidentiality of big data related to hardware and software implementations, mobile devices and wireless networks, shared resources, and shared control over monitoring systems (Ko et al. 2010). Breaches of provider medical data occur frequently with about 90 % of health care providers reporting at least one data breach over the last 2 years in an international study in 2015 (Experian 2015). The use of commercial apps for monitoring also complicates privacy issues. Patients may incorrectly assume that national medical privacy regulations apply to data collected and processed by non-providers (Glenn and Monteith 2014b). Patient posting of private medical data online, such as in support groups, is another complication, and online data cannot really be deleted due to the distributed and redundant storage of Internet data (President’s Council 2014). Preserving privacy in big data research is particularly difficult, since this often includes multiple international collaborators, and data are copied and shared around the world. The legal framework for medical privacy varies among countries (Dove and Phillips 2015).

### Ethical considerations

There is disagreement about the importance of informed consent for big data research (Rothstein 2015), with some wanting to ease regulations (Larson 2013). The consent process is of particular importance for bipolar disorder due to the highly sensitive information in the EMR (Clemens 2012), and since some patients have cognitive impairment (Daglas et al. 2015).

De-identification is frequently used to protect individual privacy. De-identified data are not covered by US federal privacy laws and are sold commercially. Yet the general public cares about using de-identified data without consent (McGraw 2013), and about the specific purpose for secondary use (Grande et al. 2013). The released data may be vulnerable to re-identification since current de-identification methods are inadequate for high-dimensional data (Narayanan et al. 2016). There is a growing confluence of the interests of academic and commercial organizations in big data projects, leading to questions about ownership of the data and any benefits created, and about disposition of data if a firm goes out of business or is purchased.

In countries without a national health service, predictive models of costs may increase coverage disparities of vulnerable groups (Wharam and Weiner 2012). Predictive models being developed by commercial, non-medical companies can create ethical conflicts (Glenn and Monteith 2014a). For example, privacy and non-discrimination laws in the US that impact decisions about credit, employment, or housing do not prohibit discrimination against the predisposition of disabilities (Horvitz and Mulligan 2015).

### Unreasonable expectations for predictive models

The expectations of the general public regarding predictive models may be inappropriate. People are familiar with personalized recommendations from Netflix or Amazon, search results from Google, and advertising on Apple and Android smartphones. These predictive models are based solely on the available data, are unconnected to causal inference and underlying mechanisms, and focus on predicting the present rather than the future (Hand 2013; Curtis 2014). The validity of predictive models in business is judged by increased overall sales and profits, not by accuracy of the prediction for individual customers (McAfee et al. 2012).

Physicians may also have unrealistic expectations for models that predict behavior based on big data. Big data is non-sampled, and from sources with a purpose other than statistical inference (Horrigan 2013). Data that are created and collected by humans reflect physical place and culture, and contain hidden biases (Pope et al. 2014, Crawford 2013). More data does not necessarily improve predictions over those made using smaller datasets as data must be relevant to the question at hand (Monteith et al. 2015; Guszcza and Richardson 2014). Big data is also without context (Boyd and Crawford 2012; Bilton 2013). Furthermore, malware or denial of service attacks occur frequently, change overall Internet behavior patterns, and further complicate interpretation of human behavior (NRC 2013). Predictive models can be wrong as shown repeatedly with Google Flu (Lazer et al. 2014a, b). Predictive models in medical and related settings can be inconsistent and biased (Singh et al. 2014), have little clinical impact (Hochster and Niedzwiecki 2016), and may be most appropriate for health policy and risk stratification rather than individual risk prediction (Harris et al. 2015; Wray et al. 2013; Wharam and Weiner 2012).

### Analytical challenges

In the future, data from all provider and patient sources will be integrated, creating massive datasets for analysis. Massive datasets have issues of scale, heterogeneity, multidimensional complexity, error handling, privacy, provenance, and many types of biases (NRC 2013; Monteith et al. 2015). If analysis of big data is based on the classical methods, underlying assumptions are likely to be violated. Researchers with different backgrounds tend to have different perspectives on data analysis, using either statistical (model-based focus on variability) or algorithmic (data mining for patterns and rules) (NRC 2013; Mahoney et al. 2008) techniques. New algorithms for big data are combining the complementary strengths of both approaches.

Human judgment is an absolutely critical component of big data analysis (Wyss and Stürmer 2014; NRC 2013). To optimize the studies of big data for bipolar disorder, participation of those with expertise in psychiatry is required throughout the analytical process, such as for parameter selection and exclusion, interpretation of results, and hypothesis generation. For example, just as Captcha demonstrates the difference between human and machine image resolution (Datta et al. 2009), psychiatrist input is needed during the development of algorithms to interpret the use of language by those with bipolar disorder.

## Conclusions

Big data projects based on the data collected by providers in EMR, claims, registries, and active patient monitoring are providing valuable information on many aspects of bipolar disorder for research and clinical care. In the near future, data from passive patient monitoring will be available and integrated with the EMR, and diverse data sources from outside of medicine such as government financial data will be linked for research. This is only the beginning. Further on, data from genetics, other omics, and imaging will also be integrated with the EMR, and lead to new levels of understanding and improvement in routine care. Many significant challenges remain for big data projects, and the active collaboration of psychiatrists is required throughout the analytical process. Big data will provide the basis for transforming the understanding and management of bipolar disorder.
